# Realization of Plasmonic Microcavity with Full Transverse and Longitudinal Mode Selection

**DOI:** 10.1038/srep27565

**Published:** 2016-06-08

**Authors:** Ju Liu, Yue-Gang Chen, Lin Gan, Ting-Hui Xiao, Zhi-Yuan Li

**Affiliations:** 1 Laboratory of Optical Physics, Institute of Physics, Chinese Academy of Science, P. O. Box 603, Beijing 100190, China; 2 Department of Physics, Guizhou University, Guiyang, 550025, China

## Abstract

Surface plasmon polaritons (SPPs) manipulation is of vital importance to construct ultracompact integrated micro/nano-optical devices and systems. Here we report the design, fabrication, and characterization of a SPP microcavity with full transverse and longitudinal mode selection and control on the surface of gold film. The designed microcavity supports the fundamental and first-order transverse modes of Gaussian mode beam with controllable longitudinal modes, respectively. The transverse mode is determined by two holographic mirrors made from deliberately designed groove patterns via the surface electromagnetic wave holography methodology, while the longitudinal mode is determined by the length of cavity. Both numerical simulations and leaky-wave SPP mode observations confirm the realization of full mode selection in the fabricated cavity. Our work opens up a powerful way to fully explore longitudinal and transverse mode control in SPP microcavities, which will be beneficial for light-matter interaction enhancement, construction of novel SPP nanolaser and microlaser, optical sensing, and optical information processing.

Surface plasmon polaritons (SPPs), which are electromagnetic waves confined to the interface between metal and dielectric^1^,^2^, have many unique properties and a broad range of potential applications^3^,^4^,^5^,^6^,^7^. Recently, there has been a surge of fascinating theoretical and technological research interest in the manipulation of SPPs on metal surface as it is of vital importance to construct ultracompact integrated micro/nano-optical devices and systems^8^,^9^,^10^. The simplest scheme for SPPs manipulation is to build a confined channel for SPPs. For example, Bozhevolnyi et al. firstly realized channel plasmon-polariton propagation along a subwavelength metal V-shaped groove in 2005^11^. Many other subwavelength plasmonic waveguides have been proposed such as the metal-insulator-metal waveguide^12^ and slot waveguide^13^, which have numerous applications in waveguide bends, splitters, interferometers, and logic devices^14^,^15^.

A more flexible scheme aims to manipulate the wavefront of plasmonic waves relies on the diffraction and interference of surface waves evolving within planar metal regions surrounded by purposely distributed scatterers. The work of Alberto *et al.* is a representative example of this idea, in which an array of holes, creating a so-called “near-field optical phase antenna”, refocus the source of the SPPs^16^. Fang demonstrated that SPPs can be focused by using Ag-column arrays and the plasmon focus size, shape, and strength can be manipulated by symmetry broken nanocorrals under linearly polarized illumination^17^,^18^. Patterning metallic surfaces to utilize the scattering of SPPs has attracted much attention^19^,^20^,^21^,^22^ since Lezec showed that by creating a periodic texture on the exit side of a single aperture in a metal film, the transmitted light can emerge as a beam with a small angular divergence^19^. The advantage is that these structured components have a subwavelength scale and the total structure can be fabricated in a small area on a flat metallic surface^23^. The realization of general functionalities of wavefront shaping can be fulfilled by complicated holographic groove patterns, which can be directly determined and designed by using the surface electromagnetic wave holography (SWH) methodology invented by us^24^. The SWH methodology has been successfully used in single-point focusing, single-direction beam collimation, two-points focusing, complex-pattern formation in free space, and plasmonic lenses^24^,^25^,^26^,^27^,^28^. In this paper we will use this methodology to deal with SPP resonance microcavity with unique mode characteristics.

Plasmonic micro/nano-cavity is critical for realizing plasmon nanolasers that allows confinement and enhancement of optical wave radiated from gain materials into deep-subwavelength nanoscale space^29^,^30^. Many plasmonic cavities of different geometric configurations have been reported^29^,^30^,^31^,^32^,^33^,^34^,^35^,^36^. Lights are confined in the optical cavity through back and forth reflection, and form resonance for special discrete frequencies^37^,^38^,^39^. This resonance leads to high photon density in cavities for strong stimulated emission. The discrete resonant frequencies correspond to different longitudinal modes and transverse modes, depending on the longitudinal length of the cavity and transverse shape of mirrors, respectively. In above SPP cavities, the cavity mode is dominated by the overall geometric configuration of the cavity, which can support single or multiple plasmonic resonance modes depending on the size of the cavity. Once the geometry of plasmonic cavities is given, the resonance SPP modes will be determined either through numerical simulation or experiment observation. Yet, it is not easy to fully manipulate the modal profile, including both the longitudinal mode and the transverse mode, of SPP cavity modes, to satisfy highly diversified and flexible demands. A more challenging problem arises: Can one manage the microscale/nanoscale cavity mode in a way as powerful as the classical macroscopic laser cavity does to laser light to fulfill some predesignated desire? And, can one go around the great obstacle of this seemingly standard optical inverse-problem and find a simple and straightforward way to solve this challenge?

In this paper, we report successful design and fabrication of a novel type of SPP microcavity where the longitudinal mode and the transverse mode are decided by the cavity length and the shape of the mirrors, respectively. In particular, we realize SPP microcavities supporting the fundamental Gaussian mode and first-order Gaussian mode, respectively. The uniqueness and aslo the secrecy of mode selection lie in the construction of special mirrors of cavity by using the SWH methodology. The mirror can shape as desire the reflection amplitude and phase of SPPs from the mirror and their wavefront of transport on metal surface, forming a specific transverse mode as designated within the cavity.

## Materials and Methods

**Figure 1:**
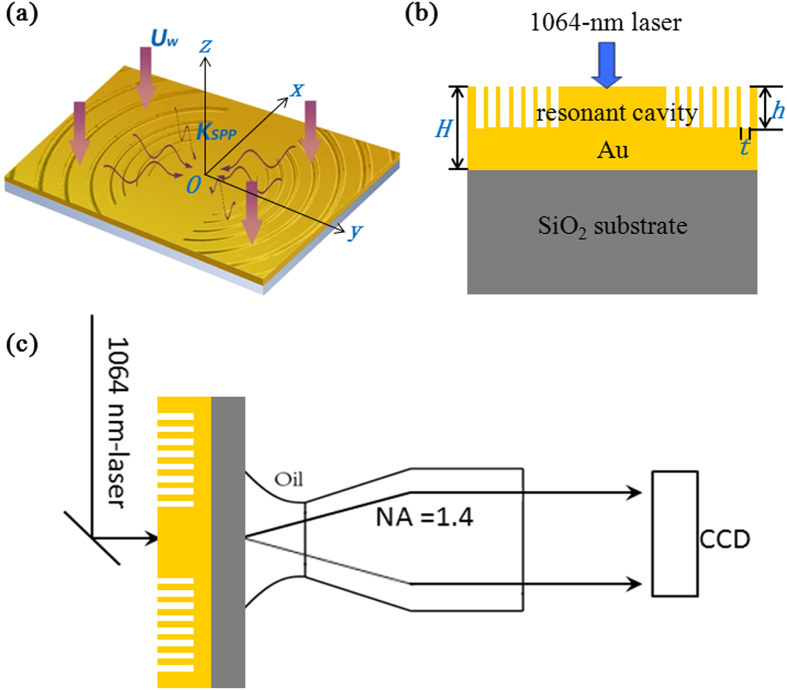
Schematic illustration of SPP microcavity with mode selection. (**a**) 3D perspective view and (**b**) 2D cross sectional picture of the cavity structure. The confocal microcavity is composed of a planar Au thin film supporting SPPs in the center part and two identical groove-pattern holographic mirrors reflecting SPPs to form steady cavity modes with both controllable transverse and longitudinal mode. The depth and the width of the groove are *h* and *t*, respectively, and the thickness of the gold film is *H*. (**c**) Experimental setup of leaky-mode observation of SPP modal profile. A 1064-nm-laser illuminates on the grooves of the gold film, a 100× NA1.4 Olympus oil lens is used to monitor the SPP field distributions and a charge-coupled device (CCD) to capture the images.

According to Boyd and Gordon, a classical Gaussian laser beam is produced through reflecting wave back and forth in a confocal cavity consisting of two appropriately designed reflection mirrors^37^,^38^,^39^. The question is whether this concept works equally well to SPPs and how to form a confocal cavity for SPPs on metal surface. The key is to design and construct an appropriate reflection mirror for SPPs to shape as desire the reflection amplitude and phase and their wavefront of transport on metal surface. We find that such a mirror can be built from a holographic groove pattern etched into metal surface, and its morphology can be determined easily by using the methodology of surface wave holography (SWH)^24^,^25^,^26^,^27^,^28^. Each holographic groove pattern will reflect the SPP wave with desired wavefront, and two well-designed groove-pattern holographic mirrors are combined to reflect SPP waves back and forth similar to mirrors in the Fabry-Perot cavity of conventional lasers. The SPP wave with a special wavefront is self-consistent in its wavefront evolution during the multiple reflections and can be confined in the cavity by the two holographic mirrors as depicted in Fig. 1. This special wavefront corresponds to the desired transverse mode of the SPP microcavity. Guided by theoretical evaluation, we realize the designed holographic mirror SPP microcavity on a gold thin film using the focused-ion beam (FIB) lithography. The gold film is deposited on SiO_2_ substrates by magnetron sputtering. A normally incident 1064-nm laser light illuminates the holographic mirrors and excites the SPP cavity mode with designated transverse and longitudinal modal profiles.

## Results and Discussion

### SPP microcavity supporting fundamental Gaussian mode

We first consider a SPP microcavity that supports a fundamental Gaussian SPP beam as the transverse mode at the wavelength of 1064 nm to illustrate the design scenario. Following the idea of confocal cavity, we take as the key to construct a mirror that can couple the free space illumination light into a SPP focusing spot on the metal surface. The SWH methodology is used to determine the groove-pattern morphology of the holographic mirror. According to this methodology^24^,^25^,^26^,^27^,^28^, the holographic pattern is directly connected with the interference pattern on the metal surface between the object wave *U*_*O*_, which is the SPP wave focusing inwardly to the designated spot, and the reference wave *U*_*W*_, which is simply the normally incident plane wave. Mathematically, *U*_*O*_ is a cylindrical wave with the complex amplitude 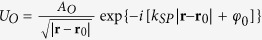. It carries the object information, transports on the metal surface (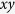 plane with *z* = 0), and focuses at the position 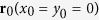. Here 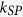 is the wave vector of SPPs on metal surface with 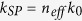, where 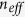 is the effective refractive index of SPPs, *k*_0_ is the wave vector in vacuum and *A*_*o*_ is the amplitude of the object wave. The reference wave is a plane wave with *U*_*W*_ = *A*_*W*_ exp[−*ik*_0_*z*], where 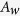 is the amplitude. The wave propagates along the *z* axis and the polarization direction is along the *y* axis. At the considered wavelength *λ*_0  _= 1.064 μm, the dielectric constant of gold is *ε*_*m*_ = −48.75 + 3.6*i*, and *k*_*SP*_ can be easily calculated.

The object wave interferes with the reference wave in a broad area on metal surface. Yet, we confine the groove-pattern holographic mirror within a finite space of −10 μm < *x *< 10 μm and 8 μm < *y* < 28 μm, which is 8 μm in distance from the object point. The hologram is made from a series of 0.24 μm wide and 0.3 μm deep grooves etched into gold film at the positions of the interference pattern maximum. In practice, we divide the hologram into a number of 40 × 40 nm^2^ pixels (corresponding to a 501 × 501 matrix). We first find all the pixels with local maximums, then etch 6 pixels into the gold film around each of these pixels along the *y*-axis, forming a series of 240 nm wide groove centered around the local maximum curves of the interference pattern. This is the final SWH pattern written via physical etching into the gold film.

**Figure 2:**
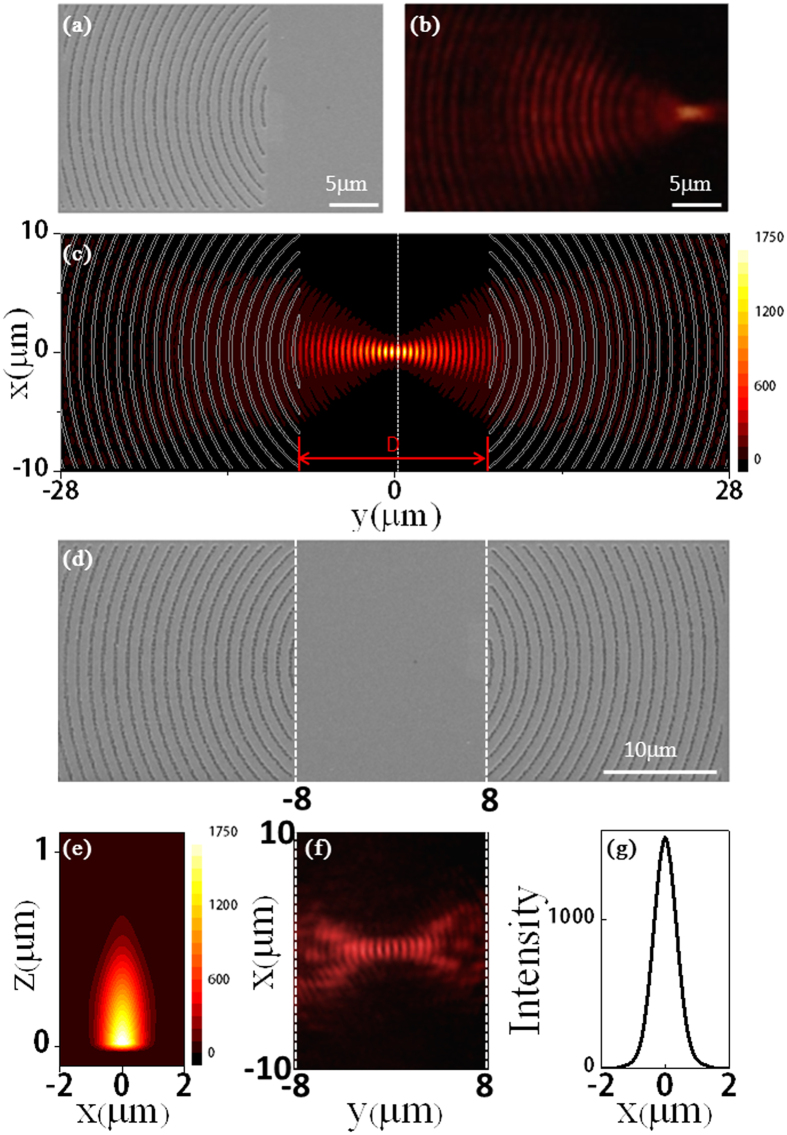
Design and fabrication of SPP microcavity supporting a fundamental Gaussian mode. (**a**) The SEM picture of the fabricated groove-pattern holographic mirror to focus SPP wave to a spot on the surface of gold thin film. (**b**) Experimental images of focusing a normally incident plane wave from free space into a SPP spot on the gold surface. (**c**) Morphology of the designed microcavity structure and calculated intensity distributions on the metal surface. The intensity distributions correspond to the modular square of electric field. The calculated field profile (**e**) in the 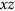 plane with *y *= 240 nm and (**g**) along the *x*-axis with *y* = 240 nm and *z* = 0. (**d**) SEM picture of the fabricated SPP concave microcavity. Each holographic mirrors has a size of 20 × 20 μm^2^, and the cavity length is 16 μm. (**f**) Leaky-mode observation image of the SPP field pattern in the concave microcavity. Both simulations and experiments show formation of a fundamental Gaussian SPP mode confined within the microcavity and at the surface of gold thin film.

When a reconstruction wave same to the reference wave illuminates on this holographic mirror, a focus of SPP should be expected^24^,^25^,^26^,^27^,^28^. To confirm this experimentally, we transfer the designed groove patterns, approximately 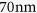 in depth to a gold film with a thickness of 90 nm. The scanning electron microscopy (SEM) image of the fabricated holographic mirror is illustrated in Fig. 2(a), where the groove pattern resembles a set of concentric rings. We then use the leaky mode observation technology as depicted in Fig. 1(c) to monitor the field distribution of the fabricated samples. A leaky mode is a mode with its electric field decaying monotonically for a finite distance in the transverse direction but substantially maintaining its shape as it decays^40^,^41^. A 100× Olympus oil lens is used to search the gold-film sample and monitor the field distributions. The 1064-nm laser is led in from the opposite side of the imaging objective. Images are captured by a charge-coupled device connected to a computer. Notice that in experiment the thickness of gold thin film and the depth of grooves are smaller than in design because thinner film allows for easier leakage and more efficient optical observation of SPP mode. However, the SPP modal profile remains the same on both values of film thickness and groove depth [Supplementary Information, Figure S1]. As clearly shown in Fig. 2(b), the incident wave is scattered by the grooves to excite the SPPs. Then the SPP beam is focused at a spot 8 μm in distance from the groove region, right at the position of the designated object point.

Obviously the grooves pattern as shown in Fig. 2(a) can serve as the holographic mirror to form a desirable confocal SPP microcavity with its geometry illustrated in Fig. 2(c) and support a self-consistent fundamental Gaussian mode. To confirm this, we carry out numerical simulation by using the three-dimensional (3D) finite-difference time-domain (FDTD) method. The calculated SPP intensity distributions (normalized to the normally intensity of incident plane wave) at the metal surface are shown in Fig. 2(c) for *λ* = 1.064 μm when *D* = 16 *μ*m. The depth and width of the grooves are 0.3 μm and 0.24 μm, respectively. In Fig. 2(c) a series of circular rings appear within the cavity, indicating formation of standing waves originating from the resonance of SPP wave in the cavity. The SPP modal profile follows the fundamental Gaussian beam distribution. At the brightest spot of the beam (the white dotted line), the intensity distribution in the 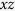 cross-sectional plane with *y* = 240 nm and along the *x*-axis with *y* = 240 nm and *z* = 0 are calculated and shown in Fig. 2(e,g), respectively. The field profile in the *x*-axis direction agrees very well with the fundamental Gaussian function and the SPP beam is strongly confined at the metal surface. As the holographic mirrors can efficiently transform the incident light into SPPs and store the energy within the cavity, the maximum SPP intensity can reach 1752 times the incident intensity.

Experimentally we transfer the designed groove patterns (70 nm in depth) to the gold film (90 nm thick) to form the desired confocal SPP microcavity, with the SEM image displayed in Fig. 2(d). The area of the groove region of each holographic mirror is 20 μm × 20 μm, and the cavity length is 16 μm. In this confocal microcavity, the two groove patterns couple the incident light to SPP wave focusing at the same point in the cavity center from two sides. The SPP waves propagating on the metal surface are reflected back and forth by the mirrors and confined in the cavity. According to ref. [38, 39], the fundamental Gaussian mode can be generated. We again use the leaky mode observation to monitor the field distributions of this confocal microcavity. The result is illustrated in Fig. 2(f) within the cavity region with the scale intentioanlly matched with the SEM picture in Fig. 2(d). Standing wave patterns are clearly observed and the overall field pattern is consistent with the field profile calculated by FDTD simulation [Fig. 2(c)], confirming experimentally the formation of SPP fundamental Gaussian mode in the designed and fabricated SPP microcavity. To describe the performance of the microcavity, we calculate the mode volume of the cavity, which is defined as 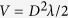. Here, *D* = 16 μm and *λ* = 1.064 μm, and the mode volume is as small as 136 μm^3^.

### SPP microcavity supporting first-order Gaussian mode

**Figure 3:**
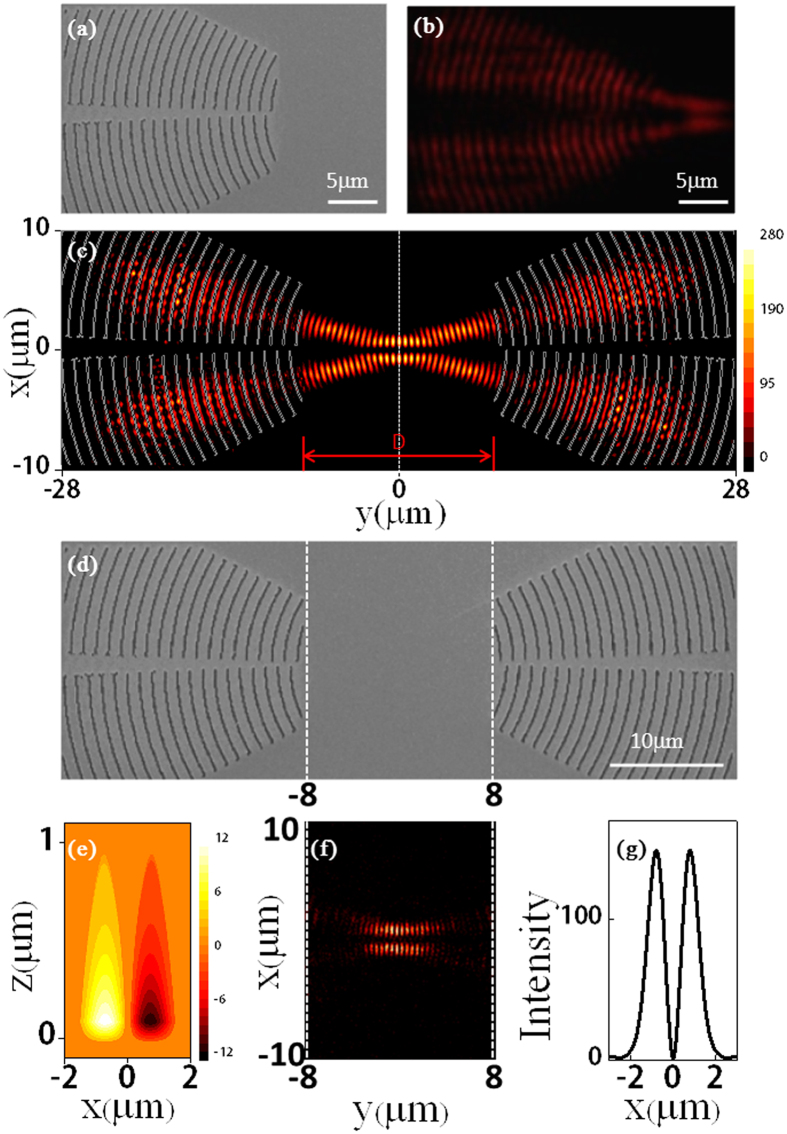
Design and fabrication of SPP microcavity supporting a first-order Gaussian mode. (**a**) The SEM picture of the fabricated groove-pattern holographic mirror to focus SPP wave to two spots on the surface of gold thin film. (**b**) Experimental images of focusing normally incident plane wave from free space into two SPP spots on the gold surface. (**c**) Morphology of the designed microcavity structure and calculated SPP intensity distributions on the metal surface of *xy* plane with *z* = 0. The intensity distributions correspond to the modular square of electric field. The calculated (**e**) *z* component of electric field profile in the 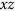 plane with *y* = 0 and (**g**) the modular square of electric field profile along the *x*-axis with *y* = 0 and *z* = 0. (**d**) SEM picture of the fabricated SPP concave microcavity. Each holographic mirror has a size of 20 ×20 μm^2^, and the cavity length is 16 μm. (**f**) Leaky-mode observation image of the SPP field pattern in the concave microcavity. Both simulations and experiments show formation of a first-order Gaussian SPP mode confined within the microcavity and at the surface of gold thin film.

We go further to consider a more complicated SPP microcavity that supports the first-order Gaussian SPP beam transverse mode at the wavelength of 1064 nm. The SWH methodology is again used to design the groove-pattern holographic mirror for this microcavity. Different from the case of fundamental Gaussian mode, now each mirror is expected to focus the normally incident light from free space into two focal SPP points with equal amplitude and 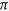 phase contrast. The object waves are set as two cylindrical waves 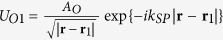  and 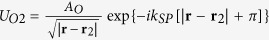, which focus inwardly at two designated positions with 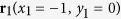 and 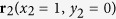, respectively. The reference wave *U*_*W*_ is still a plane wave with 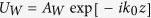. The holographic mirror is formed by etching the grooves into metal surface at the maximum intensity positions of the interference pattern formed between *U*_*o*_ and *U*_*W*_ The SEM picture of the fabricated holographic mirror is illustrated in Fig. 3(a). The groove patterns look like two sets of concentric rings with relative displacement along the radial direction. Figure 3(b) illustrates the leaky mode observation of field distributions of this holographic mirror illuminated by a normally incident 1064-nm laser. Two focal SPP spots are observed right at the positions deignated by the theoretical design.

When two above identical groove-pattern holographic mirrors are set opposite to each other with a distance *D*, a confocal SPP cavity as depicted in Fig. 3(c) is formed. The distance 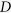 can be adjusted to select the longitudinal cavity mode. The SPP wave with the first-order Gaussian beam wavefront is self-consistent during the multiple back-and-forth reflections from the two mirrors and thus a first-order Gaussian transverse mode can form within this deliberately designed confocal SPP cavity. We can see from the FDTD simulated SPP field intensity on the metal surface as displayed in Fig. 3(c) that interference fringes (a signature of standing wave formation) appear within the cavity and the SPP beam is divided into two parts laterally (*x*-axis) when it transports along the cavity axis (*y*-axis) (a signature of first-order Gaussian beam formation). The maximum intensity, 152 times of the incident light intensity, occurs at the waist of the SPP first-order Gaussian beam as marked by the dotted line (*y* = 0). Figure 3(e) illustrates the calculated *z* component of electric field intensity in the vertical *xz* plane at *y* = 0. The exponentially decay of field is observed, indicating formation of a true SPP wave mode within the cavity. Besides, the negative and positive signs are clearly recognized, indicating the *π* phase difference between upper and lower antisymmetric parts of the SPP beam. Figure 3(g) shows that the calculated lateral field intensity distribution, i.e., along the *x*-axis with *y* = *z* = 0, is consistent with the spatial profile of the first-order Gaussian curve. All these features indicate that the designed confocal microcavity with complicated holographic mirrors indeed supports a first-order Gaussian transverse mode of SPP beam.

We experimentally fabricate the designed SPP microcavity by directly transferring the designed groove patterns to a gold film by using FIB lithography. The gold film is about 90 nm thick and the grooves are approximately 70 nm deep. The SEM image of the fabricated confocal SPP microcavity on the metal surface is displayed in Fig. 3(d), where the configuration and position of the two complicated holographic mirrors can be clearly identified. The experimental observation of SPP field pattern is illustrated in Fig. 3(f), which is well consistent with the theorerical prediction as shown in Fig. 3(c), confirming experimentally that the designed microcavity does support a first-order Gaussian transverse mode of SPP beam.

### Control of longitudinal mode

**Figure 4:**
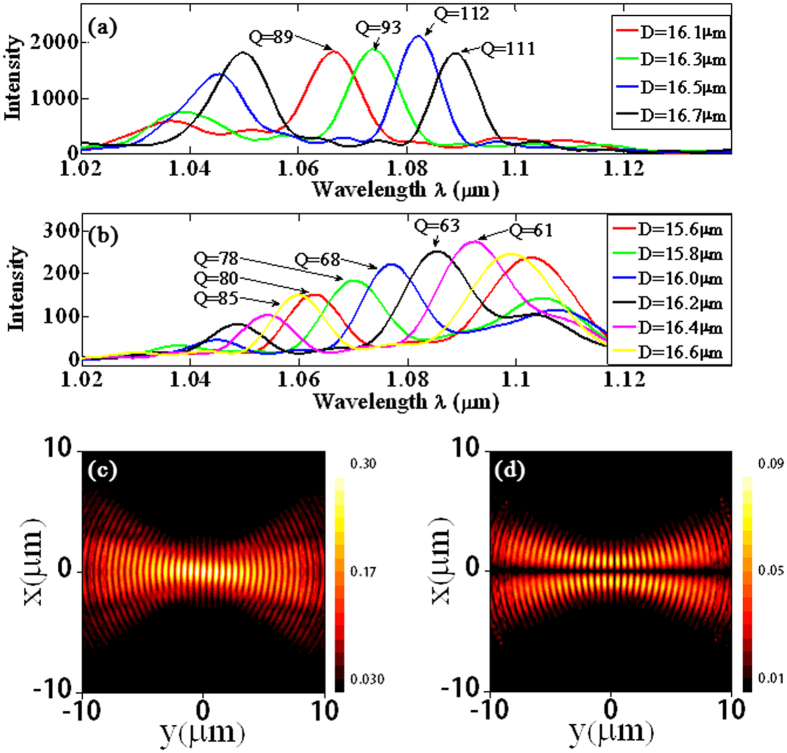
Quality factor and modal profile of SPP microcavity with mode selection. (**a**,**b**) The calculated intensity spectra at different cavity lengths *D* for the fundamental and first-order Gaussian mode SPP microcavity, respectively. (**c**,**d**) The calculated modal profile of the fundamental and first-order Gaussian SPP mode excited by a dipole source after 70 periods of light oscillation, respectively.

The resonant cavity mode of laser cavity is characterized not only by its transverse mode, but also by the longitudinal mode. Whereas the transverse mode in a SPP microcavity, e.g., the fundamental and first-order Gaussian mode, must be manipulated by complicated holographic mirrors that can only be realized via deliberate theoretical design, the manipulation of longitudinal modes is much simpler: changing the cavity length suffices to work. To show this, we use the 3D FDTD method to investigate the influence of the cavity length on the longitudinal mode. In the simulation of the fundamental Gaussian mode SPP cavity, a monitor is set at the center of cavity to record SPP fields under excitation of light at various wavelengths. The calculated intensity spectra for different cavity length *D* are shown in Fig. 4(a). The depth and width of grooves are 0.3 μm and 0.24 μm, respectively. When *D* = 16 μm, two peaks appear in the considered wavelength range, with the short-wavelength peak located at *λ* = 1.037 μm and the long-wavelength peak located at *λ* = 1.066 μm. Obviously these two peaks correspond to two longitudinal modes. When *D* changes from 16.1 to 16.7 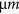, the longitudinal modes continuously red shift. When *D* = 16.5 μm, the peak intensity at *λ* = 1.082 μm reaches maximum, then decreases when *D* further increases. The spectral peaks only appear in the range from 1.03 to 1.15 μm, as this is the spectral width for the holographic mirrors to efficiently confine the SPPs on metal surface. The calculated quality (Q) factor of the resonant peaks for different cavity lengths is explicitly noticed in Fig. 4(a). One can see that the cavity with *D* = 16.5 μm has the maximal Q-factor.

The calculated intensity spectra for different cavity length *D* of the first-order Gaussian mode SPP cavity are shown in Fig. 4(b). For *D* = 15.6 μm and 0.3 μm deep grooves, two peaks appear in the considered spectral range. The short-wavelength peak has a Q-factor of 85 and is located at *λ* = 1.062 μm, while the long-wavelength peak is located at *λ* = 1.103 μm. With *D* increasing, both peaks red shift. When *D* = 16.5 μm, the short-wavelength peak red shifts to *λ* = 1.091 μm and reaches maximum in intensity, then decreases with 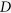 further increasing. Comparison between Fig. 4(a,b) shows that the fundamental Gaussian mode SPP cavity has a slightly higher Q-factor and a much higher resonant peak magnitude than the first-order Gaussian mode SPP cavity. This might be attributed to the higher coupling efficiency and reflection coefficient of the holographic mirrors in the former cavity, which are made from concentric rings of grooves, than those in the latter cavity, which are made from displaced and broken concentric rings of grooves. Dispite these differences, it is clear that one can efficiently control the transverse and longitudinal modes of SPP cavities.

In the above design of SPP cavity, the light source to excite SPP cavity mode is an external plane wave light. Yet, in practical performance of laser cavity, either classical macroscopic cavity, or current SPP cavity, or other nanocavity, the light source comes from gain medium that is integrated with the cavity. The gain medium, when pumped by external sources, will radiate at the cavity resonant wavelength and then amplify the cavity mode. To verify that the designed SPP microcavities can generate desirable SPP modes effectively under these practical working conditions, we excite the resonant modes by placing a dipole source within the cavities to model gain medium radiation. The orientation of the dipole source is along the *z*-axis and the dipole emits optical pulses centered at *λ* = 1.064 μm with a duration of 6.05 fs. They transport within the cavity with multiple back-and-forth reflections by the holographic mirrors accompanied with a slight round-trip attenuation in the cavity. After a long time, only the cavity resonance modes survive and the consequent field pattern can be used to identify the mode feature. In the cavity shown in Fig. 2(c), the dipole is located at 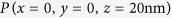. SPP waves are excited by the dipole and reflected by the holographic mirrors. Figure 4(c) shows the calculated field pattern after 70 periods delay. Even after such a long time, the field pattern remains as the fundamental Gaussian mode, identical to the pattern in Fig. 2(c). In the cavity shown in Fig. 3(c), two dipoles with an initial 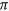 phase difference are set at the position of *P*_1_(*x* = −1 μm, *y* = 0, *z* = 20 nm) and 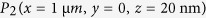. The calculated field pattern after 70 periods delay is shown in Fig. 4(d). It is also identical to the pattern in Fig. 3(c), which is a first-order Gaussian mode. The unchanged intensity profile indicates that the cavity can excite effectively the SPP mode under different means of pump source, and the mode can survive in the cavity for a long time.

According to standard macroscopic laser cavity theory^38^,^39^, the transverse modes are determined by the geometric shape of the mirror and the associate reflection wavefront of laser beam. In the above discussions we have shown both theoretically and experimentally that this general rule also holds true for SPP microcavities. However, the reflection mirrors of SPP microcavity are much more complicated in geometric configuration than their counterparts in classical laser cavity, which are some simple concave mirrors with high reflectivity. Therefore, it is no longer good to use the conventional design methodology. Instead, new concepts and methodologies must be invented and adopted to realize a complete manipulation of lasers at microscale and nanoscale, not only the threshold and power, but also the transverse and longitudinal modal profiles. Such an appeal to higher performance holds not only to current SPP lasers, but also to other types of lasers, such as distributed feedback lasers, vertical cavity surface emitting lasers, and photonic crystal lasers, all built on the semiconductor platform.

## Conclusions

In summary, we have shown easy design and realization of a plasmonic microcavity on metal surface with full transverse and longitudinal mode selection and manipulation via deliberately determined and fabricated holographic mirrors and adjustment of the cavity length, respectively. These holographic mirrors have a much more complicated geometry than their counterparts in macroscopic laser cavity. They are difficult to obtain via usual inverse-problem solutions but easy to solve by using the methodology of SWH. Although we only demonstrate fundamental and first-order transverse Gaussian mode, realization of more complicated transverse modes are feasible. A plasmonic microcavity with full mode selection and control, which represents a big step in nanoplasmonics and nanophotonics, can become a basic platform for building high Q-factor, mode selected, and tunable SPP laser, and also can find potential applications in high-performance sensing, detecting, light-matter interaction enhancement, and optical information processing. In a broader aspect, the concepts and methodologies developed here can be used to explore full mode control in other semiconductor laser cavities and optoelectronic devices.

## Additional Information

**How to cite this article**: Liu, J. *et al.* Realization of Plasmonic Microcavity with Full Transverse and Longitudinal Mode Selection. *Sci. Rep.* **6**, 27565; doi: 10.1038/srep27565 (2016).

## Supplementary Material

Supplementary Information
