# Supplementary material for: Realization of Plasmonic Microcavity with Full Transverse and Longitudinal Mode Selection
Source: Sci Rep. 2016 Jun 8;6:27565. doi: 10.1038/srep27565 (PMC4897782; doi:10.1038/srep27565)
Supplement: Supplementary Information [file srep27565-s1.doc]

**Realization of Plasmonic Microcavity with Full Transverse and Longitudinal Mode Selection**

**Ju Liu^1‡^, Yue-Gang Chen^2‡^, Lin Gan^1‡^, Ting-Hui Xiao^1^, and Zhi-Yuan Li^1*^**

**^1^ *Laboratory of Optical Physics, Institute of Physics, Chinese Academy of Science, P. O. Box 603, Beijing 100190, China***

**^2^*Department of Physics,*** ***Guizhou University, Guiyang, 550025, China***

***Corresponding author. Email address:** [**lizy@aphy.iphy.ac.cn**](mailto:lizy@aphy.iphy.ac.cn)

**‡These authors contributed equally.**

In the simulation, the thickness of gold film and the depth of grooves are $2 \text{μm}$ and$0.3 \text{μm}$, respectively, whereas in experiment the thickness of gold thin film and the depth of grooves are smaller than in design because thinner film allows for easier leakage and more efficient optical observation of SPP mode. As shown in the supplementary figure S1, the thickness of gold film of (a) and (b) are $0.09 \text{μm}$and $2 \text{μm}$, and the depth of grooves are (a) $0.07 \text{μm}$and (b) $0.3 \text{μm}$, respectively. However, the SPP modal profile remains the same on both values of film thickness and groove depth.


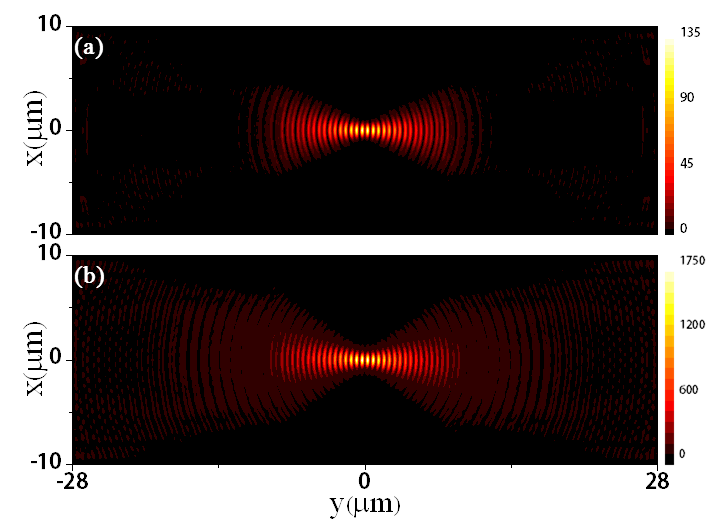


**Figure S1. Comparison of calculated SPP cavity modal profile for two values of film thickness and groove depth**. The thickness of gold film and the depth of grooves are $0.09 \text{μm}$ and $0.07 \text{μm}$, respectively in panel **a** and $2 \text{μm}$ and $0.3 \text{μm}$, respectively in panel **b**.
